# Building-Related Symptoms among Office Employees Associated with Indoor Carbon Dioxide and Total Volatile Organic Compounds

**DOI:** 10.3390/ijerph120605833

**Published:** 2015-05-27

**Authors:** Chung-Yen Lu, Jia-Min Lin, Ying-Yi Chen, Yi-Chun Chen

**Affiliations:** 1 School of Post-Baccalaureate Chinese Medicine, China Medical University, Taichung 404, Taiwan; E-Mail: u100030082@cmu.edu.tw; 2 Research Center for Traditional Chinese Medicine, Department of Medical Research, China Medical University Hospital, Taichung 404, Taiwan; 3 Institute of Environmental Health, National Taiwan University, Taipei 100, Taiwan; E-Mail: jmlin@ntu.edu.tw; 4 Institute of Environmental and Occupational Health Sciences, National Yang-Ming University, Taipei 100, Taiwan; E-Mail: sickle0312@gmail.com; 5 Department of Health Management, I-Shou University, Kaohsiung 824, Taiwan

**Keywords:** carbon dioxide, indoor air quality, sick-building syndrome, volatile organic compounds

## Abstract

This study investigated whether sick building syndrome (SBS) complaints among office workers were associated with the indoor air quality. With informed consent, 417 employees in 87 office rooms of eight high-rise buildings completed a self-reported questionnaire for symptoms experienced at work during the past month. Carbon dioxide (CO_2_), temperature, humidity and total volatile organic compounds (TVOCs) in each office were simultaneously measured for eight office hours using portable monitors. Time-averaged workday difference between the indoor and the outdoor CO_2_ concentrations (dCO_2_) was calculated as a surrogate measure of ventilation efficiency for each office unit. The prevalence rates of SBS were 22.5% for eye syndrome, 15.3% for upper respiratory and 25.4% for non-specific syndromes. Tiredness (20.9%), difficulty in concentrating (14.6%), eye dryness (18.7%) were also common complaints. The generalized estimating equations multivariate logistic regression analyses showed that adjusted odds ratios (aORs) and 95% confidence interval (CI) per 100 ppm increase in dCO_2_ were significantly associated with dry throat (1.10, 95% CI = (1.00–1.22)), tiredness (1.16, 95% CI = (1.04–1.29)) and dizziness (1.22, 95% CI = (1.08–1.37)). The ORs for per 100 ppb increases in TVOCs were also associated with upper respiratory symptoms (1.06, 95% CI = (1.04–1.07)), dry throat (1.06, 95% CI = (1.03–1.09)) and irritability (1.02, 95% CI = (1.01–1.04)). In conclusion, the association between some SBS symptoms and the exposure to CO_2_ and total VOCs are moderate but may be independently significant.

## 1. Introduction

The general population spends an average 80%–90% of their time in indoor environments [1,2]. The indoor air quality has been of great concern for the relationship with sick building syndrome (SBS) symptoms among employees in the offices [3]. Individuals have SBS when they spend time indoors and their symptoms ease when away from the buildings [2,4,5,6,7]. Among the evidence, building characteristics, indoor environmental quality [8,9] and indoor air quality have been associated with SBS. Carbon dioxide (CO_2_) levels have been considered the major factor associated with SBS symptoms [10,11,12,13,14,15]. After reviewing 21 studies involving 30,000 subjects in more than 400 buildings in cities of North America, Europe and Asia, Seppänen *et al.* concluded that SBS symptoms are associated with low ventilation rates or elevated CO_2_ levels [12]. Apte *et al.*, measuring the difference between the indoor CO_2_ concentration and the outside concentration, reported that the odds ratio (OR) of respiratory symptoms may increase from 1.1 to 1.5 for per 100 ppm increase of the indoor CO_2_ concentration [13]. A recent study in Taiwan found more complaints of eye irritation and respiratory symptoms when employees were exposed to an indoor CO_2_ concentration higher than 800 ppm [15]. The CO_2_ concentration reflects the accumulation level of indoor air pollutants associated with ventilation efficiency.

Volatile organic compounds (VOCs) are common indoor air pollutants in response to both indoor and outdoor emissions and also have been the focus of SBS in etiologic studies [14,16,17,18]. VOCs may cause irritation of the eyes, skin, respiratory tract, central nervous system and viscera. Kim *et al.* found a significant association between respiratory symptoms and indoor total microbial volatile organic compound concentration [19]. A Japanese study found aldehydes and aliphatic hydrocarbons in new single-family houses were positively associated with SBS [20]. Mølhave suggested that a level of VOCs higher than 3.0 mg/m^3^ in a non-industrial indoor environment might be associated with harm to human health or comfort [21]. Smoking and other human activities increase total indoor volatile organic compound (TVOC) concentrations [22].

Although several studies have suggested CO_2_ and VOCs levels are the main SBS contributors, public concerns need more information about the association. In this study, we investigated the prevalence of non-specific complaints about SBS, upper and lower respiratory symptoms and examined the relationships between these symptoms and indoor concentrations of CO_2_ and TVOCs.

## 2. Materials and Methods 

### 2.1. Questionnaire Survey 

Employees working in 16 institutions located in high-rise buildings in Taipei city were randomly selected and invited to participate in this SBS study. We measured indoor air quality at their working places. The details of this study have been reported in previous studies [23,24]. Invitation letters explaining this study were delivered to potential participants and a total of 417 persons responded to our self-reported questionnaire survey with the required informed consent. Information obtained from the questionnaire included age, gender, education, smoking and alcohol history, medical history, and the typical SBS symptoms, specifically for nose, eyes, skin, upper and lower respiratory and general complaints. An institutional review committee approved this study.

### 2.2. Environmental Measurement 

We measured the levels of CO_2_ and VOCs using portable monitors at each office for eight office hours. Both indoor and outdoor levels of CO_2_ (Q-TRAK IAQ Model 8551, TSI Incorporated, Shoreview, MN, USA) were standardized for a wide range (0–5000 ppm) with a fine resolution of 1.0 ppm. The VOC measurements (PGM-7240, RAE SYSTEMS, Sunnyvale, CA, USA) were standardized for 102 categories of VOC with an acceptable deviation of 20 ppb. The indoor air was monitored at 1.2 m height at the center of the office without mechanical ventilation inlets or outlets. We used standard gas to calibrate the instrument including the zero point check.

### 2.3. Statistical Analysis 

The SBS symptoms were defined as participants reporting one or more selected symptoms specified in the questionnaire for at least 1–3 days per week while at work in the office in the previous month, but which improved or disappeared after work or on days without work. The SBS symptoms were identified individually and as a group. Prevalence rates of SBS symptoms for eyes (eye dryness and eye irritation), upper respiratory tract (nose itching, runny nose, stuffy nose, sneezing and dry throat), lower respiratory tract (difficulty in breathing), skin (skin dryness), and non-specific symptoms (headache, tiredness, difficulty in concentrating, irritability and dizziness) were evaluated. 

In statistical analyses, associations between SBS symptoms and selected covariates, including participants’ socio-demographic status, medical history, and indoor air pollutants were first measured and tested using Pearson’s χ^2^ test and Fisher’s exact test. The SBS symptoms were analyzed both individually and in combined categories. Generalized estimating equations (GEE) were used to accommodate multiple contralateral pairs within participants. Building parameters were considered for inclusion in GEE logistic regression models for adjustment. Table 1 lists potential covariates to be used in the initial GEE logistic regression models, including demographic and environmental variables, allergies, chemical and environmental tobacco smoking sensitivity, indoor relative humidity and temperature, speck of molds, exposure to environmental tobacco smoke, presence of carpet in the office, and new carpet, furniture and decoration, and recent painting at work. 

**Table 1:** Covariates included in all generalized estimating equations logistic regression models.

Variable	Description
Gender	0: male; 1: female
Age	0: age < 40 years; 1: age ≥ 40 years
Carpet	0: no carpet on workstation; 1: carpet on most or all workspace
Smoker	0: never or former smoker; 1: current smoker
Asthma	0: previously never diagnosed; 1: previously ever diagnosed
Nasosinusitis	0: previously never diagnosed; 1: previously ever diagnosed
Atopic rhinitis	0: previously never diagnosed; 1: previously ever diagnosed
Migraine	0: previously never diagnosed; 1: previously ever diagnosed
Dust allergies	0: previously never diagnosed; 1: previously ever diagnosed
Animals allergies	0: previously never diagnosed; 1: previously ever diagnosed
Chemical sensitivity	0: previously never diagnosed; 1: previously ever diagnosed
ETS sensitivity	0: self-reported “No”; 1: self-reported “Yes”
Exposure to ETS	0: self-reported “No”; 1: self-reported “Yes”
Using Sanitizing chemical	0: self-reported “No”; 1: self-reported “Yes”
New furniture	0: self-reported “No”; 1: self-reported “Yes”
New decoration	0: self-reported “No”; 1: self-reported “Yes”
Painting recently	0: self-reported “No”; 1: self-reported “Yes”
Working stress	0: self-reported “No”; 1: self-reported “Yes”
No social support	0: self-reported “No”; 1: self-reported “Yes”
Working time＞9 h/day	0: self-reported “No”; 1: self-reported “Yes”
Speck of molds	0: “No” recorded by sampler; 1: “Yes” recorded by sampler
Leaking	0: “No” recorded by sampler; 1: “Yes” recorded by sampler
Season	0: spring; 1: winter
dCO_2_	Hourly mean degree per 100 (ppm/100)
TVOCs_indoor_	Hourly mean degree per 100 (ppb/100)
RH	Hourly mean degree (%)
Temperature	Hourly mean degree (°C)

RH, relative humidity; ETS, environmental tobacco smoking; dCO_2_, difference between indoor and outdoor carbon dioxide concentrations; TVOCs_indoor_, indoor total volatile organic compounds concentrations.

The exposure of carbon dioxide and indoor TVOCs were considered as the major factors associated with SBS symptoms. Eight-hour averaged workday differences between indoor and outdoor CO_2_ concentrations (dCO_2_＝ CO_2_ indoors − CO_2_ outdoors) [13] was used to represent the ventilation efficiency for the office. Finally, simple and step down GEE multivariate logistic regression (MLR) models were performed using each of the SBS symptoms as the dependent variables, and CO_2_ metric (dCO_2_) and TVOCs as independent variables, controlling for covariates. The odds ratio (OR) of the symptom and corresponding 95% confidence interval (CI) were calculated. The final GEE MLR analysis was performed controlling for potential confounding factors. Data analyses and plotting were conducted using the statistical package software of SAS 8.1 (SAS Institute Inc., Cary, NC, USA) and Excel, and α was taken as 0.05.

## 3. Results and Discussion

### 3.1. Indoor CO_2_ and TVOCs Measurement 

**Figure 1:**
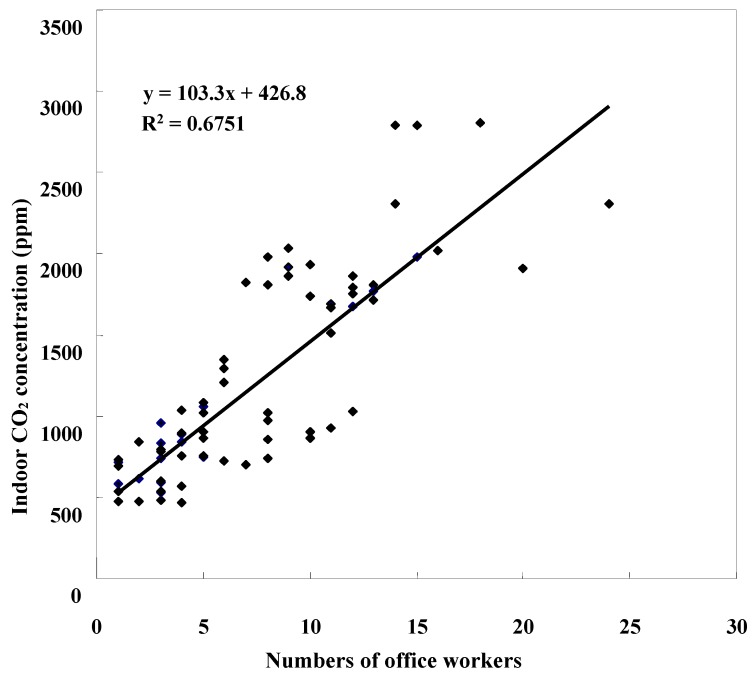
Association between numbers of office workers and indoor carbon dioxide (CO_2_) concentrations in high-rise building offices.

Figure 1 shows that the mean 8-hour CO_2_ levels at the surveyed offices in high-rise buildings, increased as the number of persons at work increased, to near 2800 ppm in an office with 25 persons at work. The average indoor temperature and relative humidity among offices were 23.6 °C and 57.3%, respectively, (Table 2). The hourly mean CO_2_ concentration of indoor (1160 ppm, SD = 604 ppm) was 2.6 times higher than that outdoors (mean = 434 ppm, SD= 60 ppm). The hourly mean TVOCs concentrations was 6.5 times greater indoors than outdoors (1190 ppb *vs.* 180 ppb).

### 3.2. Self-Reported Sick Building Syndrome Risk

Study participants were predominantly women (Table 3). High proportions of office employees reported sensitivity to tobacco (68.3%) and chemicals in the air (64.5%). SBS symptoms were prevalent: 22.5% of participants had eye symptoms, 15.3% had upper respiratory, 6.5% had lower respiratory symptoms and 25.4% had non-specific symptoms. Tiredness (20.9%), eye dryness (18.7%) and difficulty in concentrating (14.6%) were also common complaints.

**Table 2:** Measured environmental variables compared between indoor and outdoor among 87 survey offices in high-rise buildings.

Environmental Variables	Mean	SD	Range
Temperature _indoor_ (°C)	23.6	1.71	18.6–28.4
Temperature _outdoor_ (°C)	25.7	4.96	16.3–37.8
Difference ^a^	−2.13	4.48	−14.2–5.00
Relative humidity_indoor_ (%)	57.3	6.92	45.5–79.7
Relative humidity_outdoor_ (%)	60.5	13.6	34.0–81.3
Difference ^a^	−3.18	11.6	−21.1–24.2
CO_2 indoor_ (100 ppm)	11.6	6.04	4.67–28.0
CO_2 outdoor_ (100 ppm)	4.34	0.60	3.37–5.63
Difference ^a^	7.29	6.25	−1.03–23.9
TVOCs _indoor_ (100 ppb)	11.9	60.6	0.06–557
TVOCs _outdoor_ (100 ppb)	1.80	1.60	0.05–4.25
Difference ^a^	10.1	60.2	−1.46–553

CO_2_, carbon dioxide; TVOCs, indoor total volatile organic compounds; ^a^ between indoor and outdoor.

**Table 3:** Summary in prevalence of participants characteristics and sick building syndrome symptoms among office employees (n =417).

Individual Characteristics	%	Symptoms	%
Female	77.9	**Eye, any**	22.5
Age (≥40 years)	30.0	Eye dryness	18.7
Current smoker	11.3	Eye irritation	5.5
Working time >5 days/week	11.8		
Working time >9 h/day	20.9	**Upper respiratory, any**	15.3
Working stress	14.6	Nose itching	2.4
Lacking of family support	40.3	Runny nose	2.4
Asthma	3.4	Stuffy nose	6.2
Nasosinusitis	7.0	Sneezing	2.4
Atopic rhinitis	29.0	Dry throat	6.7
Migraine	17.0		
Dust allergy	24.5	**Lower respiratory, any**	6.5
Animal allergy	8.6	Difficulties in breathing	6.5
Sensitivity to tobacco smoke	68.3		
Sensitivity to chemicals in air	64.5	**Skin, any**	1.9
Exposure to ETS	15.6	Skin dryness	1.9
Using Sanitizing chemical	29.7		
Carpet in workspace	61.4	**Non-specific, any**	25.4
New furniture	4.6	Tiredness	20.9
New decoration	9.4	Difficulties in concentrating	14.6
Painting recently	5.5	Irritability	12.7
Leaking	10.1	Dizziness	7.2
Speck of molds	7.7		

After controlling for personal and environmental variables, per 100 ppm increase in dCO_2_ had significant associations with dry throat (OR = 1.10; 95% CI = 1.00–1.22), tiredness (OR = 1.16; 95% CI = 1.07–1.26), dizziness (OR = 1.22; 95% CI = 1.08–1.37) and non-specific syndrome (OR = 1.16; 95% CI = 1.04–1.29), but had a protective association with eye irritation (OR = 0.81; 95% CI = 0.67–0.98) (Table 4). Eye irritation, tiredness, dizziness and non-specific syndrome remained significant after the addition of TVOCs to the GEE MLR model. 

**Table 4:** Crude and adjusted odds ratio and 95% confidence intervals (in parentheses) for sick building syndrome symptom associated with per 100 ppm increase in difference between indoor and outdoor carbon dioxide concentrations (dCO_2_) obtained from generalized estimating equations logistic regression models (n = 417).

Sick-Building Syndrome Symptom	dCO_2_ (per 100 ppm)		
	Crude	Adjusted ^a^	Adjusted ^b^
**Eye, any**	0.99 (0.97–1.01)	1.00 (0.96–1.04)	1.00 (0.96–1.04)
Eye dryness	1.01 (0.99–1.04)	1.02 (0.99–1.06)	1.02 (0.98–1.06)
Eye irritation	**0.85 (0.73–0.99)**	**0.81 (0.67–0.98)**	**0.74 (0.59–0.93)**
**Upper respiratory, any**	1.02 (0.91–1.14)	1.04 (0.93–1.17)	0.97 (0.89–1.06)
Nose itching	1.02 (0.86–1.21)	1.03 (0.80–1.32)	1.03 (0.80–1.32)
Runny nose	0.97 (0.79–1.20)	0.92 (0.71–1.18)	0.92 (0.72–1.19)
Stuffy nose	1.03 (0.94–1.13)	1.11 (0.96–1.28)	1.07 (0.92–1.25)
Sneezing	1.04 (0.94–1.15)	0.93 (0.69–1.25)	0.52 (0.12–2.31)
Dry throat	1.03 (0.89–1.18)	**1.10 (1.00–1.22)**	1.03 (0.91–1.15)
**Lower respiratory, any**	0.99 (0.94–1.18)	1.07 (0.96–1.20)	1.05 (0.94–1.18)
Difficulties in breathing	0.99 (0.94–1.18)	1.07 (0.96–1.20)	1.05 (0.94–1.18)
**Skin, any**	1.03 (0.87–1.22)	1.04 (0.85–1.28)	1.05 (0.82–1.34)
Dryness	1.03 (0.87–1.22)	1.04 (0.85–1.28)	1.05 (0.82–1.34)
**Non-specific, any**	1.04 (0.97–1.11)	**1.16 (1.04–1.29)**	**1.13 (1.02–1.26)**
Tiredness	1.03 (0.97–1.10)	**1.16 (1.07–1.26)**	**1.14 (1.06–1.23)**
Difficulties in concentrating	1.01 (0.93–1.09)	1.09 (0.99–1.20)	1.08 (0.98–1.19)
Irritability	0.97 (0.89–1.05)	1.13 (0.95–1.35)	1.09 (0.92–1.29)
Dizziness	1.10 (0.97–1.26)	**1.22 (1.08–1.37)**	**1.20 (1.07–1.34)**

^a^ Adjusted for gender, age, smoking status, presence of carpet in workspace, new furniture, new decoration, painting recently in workspace, leaking, speck of molds, allergies, chemical and environmental tobacco smoking sensitivity, asthma, nasosinusitis, atopic rhinitis, migraine, working stress, lacking of family support, sanitizing by using chemical, exposure to environmental tobacco smoking, working time per week, working time per day, relative humidity and room temperature. ^b^ Indoor total volatile organic compounds concentrations (TVOCs) were included in the model.

Table 5 shows the associations between SBS symptoms and the per 100 ppb increase of indoor TVOCs in measured air among offices. Adjusted odds ratios per 100 ppb increase in indoor TVOCs were slightly significant for upper respiratory syndrome (OR = 1.06; 95% CI = 1.04–1.07), stuffy nose (OR = 1.01; 95% CI = 1.01–1.02), dry throat (OR = 1.06; 95% CI = 1.03–1.09) and lower respiratory syndrome (OR = 1.01; 95% CI = 1.00–1.01), non-specific syndrome (OR = 1.03; 95% CI = 1.02–1.05), tiredness (OR = 1.02; 95% CI = 1.01–1.04), angry easily (OR = 1.02; 95% CI = 1.01–1.04) and dizziness (OR = 1.01; 95% CI = 1.00–1.02). The ORs for upper respiratory syndrome, dry throat, angry easily and dizziness did not change even after adding the variable of dCO_2_ to the model for analysis.

**Table 5:** Crude, adjusted prevalence odds ratios and 95% confidence intervals (in parentheses) for sick building syndrome symptom association with per 100 ppb increase in total volatile organic compounds in indoor air (TVOCs) obtained from generalized estimating equations logistic regression models (n = 417).

Sick-Building Syndrome Symptom	TVOCs (per 100 ppm)		
	Crude	Adjusted ^a^	Adjusted ^b^
**Eye, any**	1.00 (0.99–1.00)	1.00 (1.00–1.00)	1.00 (0.99–1.00)
Eye dryness	1.00 (1.00–1.01)	1.00 (1.00–1.01)	1.00 (0.99–1.00)
Eye irritation	1.00 (1.00–1.01)	1.01 (1.00–1.01)	**1.01 (1.00–1.02)**
**Upper respiratory, any**	**1.04 (1.02–1.06)**	**1.06 (1.04–1.07)**	**1.06 (1.05–1.07)**
Nose itching	0.98 (0.92–1.04)	1.00 (0.97–1.03)	1.00 (0.97–1.03)
Runny nose	0.97 (0.90–1.06)	1.00 (0.95–1.05)	1.00 (0.96–1.04)
Stuffy nose	**1.01 (1.00–1.01)**	**1.01 (1.01–1.02)**	**1.01 (1.01–1.02)**
Sneezing	**1.01 (1.00–1.01)**	1.07 (0.86–1.33)	2.63 (0.18–38.7)
Dry throat	1.02 (1.00–1.05)	**1.06 (1.03–1.09)**	**1.06 (1.02–1.09)**
**Lower respiratory, any**	1.00 (1.00–1.01)	**1.01 (1.00–1.01)**	1.01 (1.00–1.01)
Difficulties in breathing	1.00 (1.00–1.01)	**1.01 (1.00–1.01)**	1.01 (1.00–1.01)
**Skin, any**	**1.01 (1.01–1.01)**	**1.01 (1.00–1.02)**	**1.01 (1.00–1.02)**
Dryness	**1.01 (1.01–1.01)**	**1.01 (1.00–1.02)**	**1.01 (1.00–1.02)**
**Non-specific, any**	**1.03 (1.02–1.04)**	**1.03 (1.02–1.05)**	**1.02 (1.01–1.03)**
Tiredness	**1.03 (1.02–1.04)**	**1.02 (1.01–1.04)**	**1.01 (1.01–1.02)**
Difficulties in concentrating	1.00 (1.00–1.01)	1.00 (1.00–1.01)	1.00 (1.00–1.01)
Irritability	**1.03 (1.02–1.03)**	**1.02 (1.01–1.04)**	**1.02 (1.00–1.03)**
Dizziness	**1.01 (1.01–1.01)**	**1.01 (1.00–1.02)**	**1.01 (1.00–1.01)**

^a^ Adjusted for gender, age, smoking status, presence of carpet in workspace, new furniture, new decoration, painting recently in workspace, leaking, speck of moulds, allergies, chemical and environmental tobacco smoking sensitivity, asthma, nasosinusitis, atopic rhinitis, migraine, working stress, lacking of family support, sanitizing by using chemical, exposure to environmental tobacco smoking, working time per week and working time per day, relative humidity and room temperature. ^b^ Carbon dioxide concentrations (dCO_2_) differences between indoor and outdoor were included in the model.

## 4. Discussion 

This study demonstrated that SBS symptoms experienced among employees in high-rise buildings exhibited a stronger association with CO_2_ concentrations than with TVOCs concentrations in their offices. The CO_2_ concentrations measured in office units, ranging from 467 to 2800 ppm, were much greater than that in the outdoor air (1160 *vs.* 434 ppm on average). The concentrations of CO_2_ in office buildings are primarily dependent on occupant density and ventilation rates [25].

In our study, tiredness and dizziness were associated with dCO_2_ after controlling for TVOCs; but there were no significant associations between dCO_2_ and respiratory, eye or skin symptoms. The dCO_2_ measures the difference in CO_2_ concentrations between indoor air and outdoor air. 

The CO_2_ association is somewhat consistent with the findings in the previous study [12,13,15,26,27]. Jung *et al.* found that the neuroendocrine system was associated with dCO_2_ and SBS symptoms were associated with the allostatic load level in the neuroendocrine and metabolic systems [27]. Tsai *et al.* found eye irritation and respiratory symptoms were associated with high levels of indoor CO_2_ [15]. No causal relationship between CO_2_ exposure and SBS symptoms was considered in the study by Seppänen *et al.* [12], but they did find that compared with occupants in rooms with high ventilation rates, those in rooms with less ventilation rate had ORs of SBS ranging from 1.1 to 6.0, and of respiratory complaints ranging from 1.5 to 2.0.

The dCO_2_ association with SBS symptoms in this study is different from findings in other studies in symptom sites. The study based on 41 American office buildings shows that the dCO_2_ arises per 100 ppm the ORs of having sore throat, stuffy nose, chest tightness and wheezing range from 1.1–1.5. ORs increased to 1.3–2.3 for those exposed to maximum dCO_2_ per hour greater than 250 ppm [13]. Erdmann and Apte presented a similar study with significant ORs 1.1–1.2 per 100 ppm increases in dCO_2_ for mucosal symptom and lower respiratory symptoms [26]. They considered that the cause of SBS was similar to the indoor dCO_2_. However, other pollutants were not determined in these studies. 

It has been hypothesized that office workers exposed to indoor air pollutants would have elevated risk of building-related SBS syndrome. Takigawa *et al.* reported that newly constructed hospitals with TVOC concentrations greater than 1200 g/m^3^ could induce symptoms involving not only the skin, eyes, ears, throat and chest, but also the central nervous system, autonomic system, musculoskeletal system, and digestive system with gender difference, being higher for females [28]. Saijo *et al.* recently studied newly constructed buildings and stated that toluene, butyl acetate, ethylbenzene, α-pinene, *p*-dichlorobenzene, nonanal and xylene were significantly responsible for respiratory symptoms [29]. A survey in three North European cities found the indoor concentrations of 1-octen-3-ol and 3-methylfuran increased the risk of mucosal symptoms, and the indoor levels of some microbial volatile organic compounds and formaldehyde was associated with the risk of SBS [30]. 

In this study, the average TVOC concentration was higher in indoor air than in outdoor air (1190 *vs.* 180 ppb, respectively), with a large variation among offices (range from 6 to 55,730 ppb). The offices with subsidiary printing centers have noticeably higher TVOCs concentrations (median = 504 ppb, range = 95–55,730 ppb) than other offices without printing machines (median = 100 ppb, range = 10–362 ppb). VOCs in the indoor air are probably the factor associated with human health and comfort and individuals may feel discomfort if the indoor level of VOCs is above 3 mg/m^3^ in non-industrial environments [21]. Our study found that an elevated TVOC level was associated with increased prevalence of eye dryness, eye irritation, upper respiratory syndrome of stuffy nose, sneezing and dry throat, lower respiratory syndrome of difficulty breathing, skin syndrome of skin dryness, and non-specific syndromes of tiredness, angry easily, difficulty concentrating and dizziness (ORs range 1.00–1.06 for per 100 ppb of TVOCs increase). These findings were evident in adjusted MLR models through adjustment for potential confounders.

In this study, we encountered a problem in TVOC measurement because the wavelengths available in the photo-ionization detector for detecting various VOC species are limited. The portable PID monitors used to measure TVOCs in this study was equipped with a 10.6 eV photo-ionization detector. The instrument responds to organic and inorganic gases that have an ionization potential of less than 10.6 eV, including aromatic hydrocarbons, olefins, and higher molecular weight alkanes. It does not respond to light hydrocarbons such as methane, ethane, propane, acetylene, formaldehyde and methanol. Condensing humidity in the PID will cause false values by using a cool instrument in hot and humid air. In order to prevent false signals readings, we adjusted the instrument temperature to a level similar to the room temperature before each sampling. 

Previous studies on SBS in Western countries were performed using a much larger sample size than ours [13,30]. It is interesting however to note that the risk associations found in our study are somewhat alike to their findings. It is likely our study is strengthened because we have carefully measured the 8-hour environmental risk exposures to obtain reliable data for participants. We also used adequate methods to evaluate the risk relationships. In the adjusted GEE MLR models, dCO_2_ and indoor VOCs concentrations are significantly associated with different symptoms (Table 4 and Table 5). In these models, the significant adjusted ORs per 100 ppm increase in dCO_2_ for the non-specific syndromes of tiredness and dizziness are different from those per 100 ppb increases in TVOCs for eye irritation, stuffy nose, difficulties in breathing, skin dryness, and irritability. We found that an elevated prevalence of SBS symptoms is associated with different indicators of indoor air quality.

This study applied personal responses to environmental assessments of 87 offices in eight high-rise buildings to investigate the association between indoor exposure to CO_2_ and TVOCs and SBS. It is plausible to explain the association by a biological mechanism. The ORs measured in the GEE MLR analyses show that risks of non-specific syndromes of tiredness and dizziness increase by 14% and 20%, respectively, associated with per 100 ppm increment of dCO_2_. CO_2_ levels in the blood may increase as dCO_2_ increases and cause an decrease in the oxygen saturation of the hemoglobin, resulting in oxygen starvation; which may well explain the association between dCO_2_ and complaints of tiredness and dizziness. On the other hand, the slight risks of eye irritation, stuffy nose and dry throat, difficulty in breathing, skin dryness, irritability and dizziness are associated with the volatile compounds in TVOCs. 

## 5. Conclusions 

Our study results suggest that symptoms of SBS are associated with various etiological factors. Non-specific syndromes of tiredness, difficulty to concentrate and dizziness are moderately associated with the difference of CO_2_ levels between the indoors and the outdoors. However, the risks of eye irritation, stuffy nose and dry throat, difficulty breathing, skin dryness, irritability and dizziness are slightly associated with TVOCs.
